# 9-(5-Bromo-2-hy­droxy­phen­yl)-10-(2-hy­droxy­prop­yl)-3,3,6,6-tetra­methyl-1,2,3,4,5,6,7,8,9,10-deca­hydro­acridine-1,8-dione

**DOI:** 10.1107/S1600536811013481

**Published:** 2011-04-16

**Authors:** Ali N. Khalilov, Antar A. Abdelhamid, Atash V. Gurbanov, Seik Weng Ng

**Affiliations:** a Department of Organic Chemistry, Baku State University, Baku, Azerbaijan; b Chemistry & Environmental Science Division, School of Science, Manchester Metropolitan University, England; c Department of Chemistry, University of Malaya, 50603 Kuala Lumpur, Malaysia

## Abstract

The dihydro­pyridine ring in the title compound, C_26_H_32_BrNO_4_, adopts an envelope conformation with the methine C atom representing the flap. The cyclo­hexenone rings also adopt envelope conformations. The phenolic hy­droxy group forms an intra­molecular hydrogen bond to one of the two keto O atoms. Inter­molecular weak C—H⋯O hydrogen bonding is present in the crystal structure. The hy­droxy­propyl group is disordered over two sets of sites with an occupancy ratio of 0.636 (6):0.364 (6).

## Related literature

For a related structure, see: Abdelhamid *et al.* (2011 ▶).
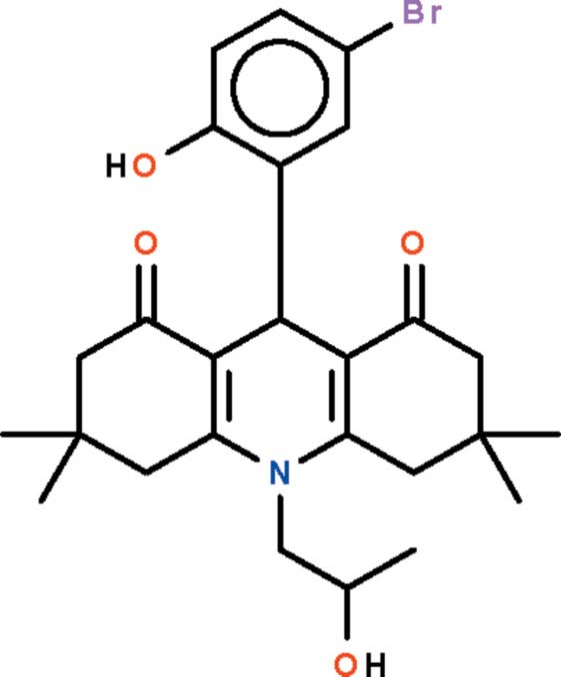


## Experimental

### 

#### Crystal data

C_26_H_32_BrNO_4_
*M* *_r_* = 502.44
Monoclinic, 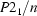

*a* = 10.6685 (4) Å
*b* = 16.8190 (5) Å
*c* = 14.1260 (5) Å
β = 106.303 (3)°
*V* = 2432.76 (14) Å^3^
*Z* = 4
Mo *K*α radiation
μ = 1.72 mm^−1^
*T* = 100 K
0.20 × 0.10 × 0.05 mm

#### Data collection

Agilent SuperNova Dual diffractometer with an Atlas detector
Absorption correction: multi-scan (*CrysAlis PRO*; Agilent, 2010 ▶) *T*_min_ = 0.725, *T*_max_ = 0.919
11939 measured reflections
5402 independent reflections
3939 reflections with *I* > 2σ(*I*)
*R*_int_ = 0.045

#### Refinement

*R*[*F* ^2^ > 2σ(*F* ^2^)] = 0.053
*wR*(*F* ^2^) = 0.135
*S* = 1.03
5402 reflections
302 parameters
24 restraints
H-atom parameters constrained
Δρ_max_ = 0.76 e Å^−3^
Δρ_min_ = −0.66 e Å^−3^

### 

Data collection: *CrysAlis PRO* (Agilent, 2010 ▶); cell refinement: *CrysAlis PRO*; data reduction: *CrysAlis PRO*; program(s) used to solve structure: *SHELXS97* (Sheldrick, 2008 ▶); program(s) used to refine structure: *SHELXL97* (Sheldrick, 2008 ▶); molecular graphics: *X-SEED* (Barbour, 2001 ▶); software used to prepare material for publication: *publCIF* (Westrip, 2010 ▶).

## Supplementary Material

Crystal structure: contains datablocks global, I. DOI: 10.1107/S1600536811013481/xu5192sup1.cif

Structure factors: contains datablocks I. DOI: 10.1107/S1600536811013481/xu5192Isup2.hkl

Additional supplementary materials:  crystallographic information; 3D view; checkCIF report

## Figures and Tables

**Table 1:** Hydrogen-bond geometry (Å, °)

*D*—H⋯*A*	*D*—H	H⋯*A*	*D*⋯*A*	*D*—H⋯*A*
O1—H1⋯O2	0.84	1.78	2.621 (4)	176
C10—H10*A*⋯O1^i^	0.99	2.54	3.417 (5)	147

Symmetry code: (i) 
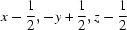
.

**Fig. 1.**
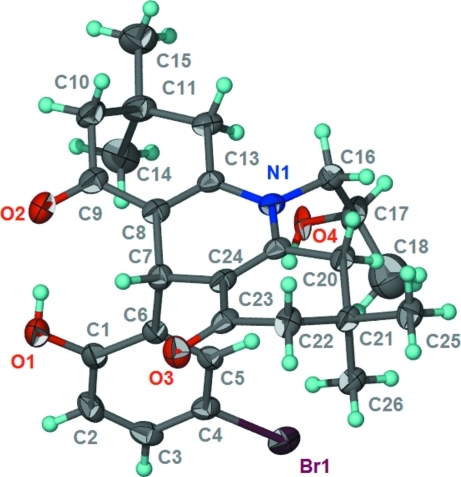
Thermal ellipsoid plot (Barbour, 2001) of C26H32BrNO4 at the 70% probability level; hydrogen atoms are drawn as spheres of arbitrary radius. The disorder is not shown.

## supplementary crystallographic information

### Comment

In an earlier study, we reported 10-(2-hydroxyethyl)-9-(2-hydroxyphenyl)-3,3,6,6-tetramethyl-1,2,3,4,5,6,7,8,9,10-decahydroacridine-1,8-dione, which was synthesized by the reaction of dimedone, salicyladehyde and 2-aminoethanol (Abdelhamid *et al.*, 2011). In the present study, we replaced 2-aminoethanol by 1-amino-2-propanol and also used a bromine-subsituted salicyladehyde to form the title analog (Scheme I). The dihydropyridine ring in the C_26_H_32_BrNO_4_ adopts an envelope conformation with the methine C atom representing the flap. The cyclohexenone rings also adopt envelope conformations with the C atoms bearing the methyl C atoms representing the flaps. The phenolic hydroxy group forms an intramolecular hydrogen bond to one of the two keto O atoms (Fig. 1).

### Experimental

5-Bromo-2-hydroxybenzaldehyde (10 mmol), 1-amino-2-propanol (10 mol) and 20 dimedone (20 mmol) were heated in pyridine (50 ml) for 5 h. The solid that was isolated from the cool solution was collected and recrystallized from ethano; m.p. 508 K.

### Refinement

H-atoms were placed in calculated positions [C–H 0.95 to 0.99, O–H 0.84 Å; *U*(H) 1.2 to 1.5*U*(C,*O*)] and were included in the refinement in the riding model approximation.

The hydroxypropyl group is disordered over two positions in respect of three of the four non-hydrogen atoms; the C atom connected to the dihydropyridine ring is ordered. The carbon–carbon distances were restrained to 1.54±0.01Å and the carbon–oxygen distances to 1.45±0.01 Å. The temperature factors of the primed atoms were set to those of the unprimed atoms, and the anisotropic temperature factors were restrained to be nearly isotropic. The disorder refined to a 63.6 (1): 36.4 ratio.

### Crystal data

**Table:**

C_26_H_32_BrNO_4_	*F*(000) = 1048
*M**_r_* = 502.44	*D*_x_ = 1.372 Mg m^−^^3^
Monoclinic, *P*2_1_/*n*	Mo *K*α radiation, λ = 0.71073 Å
Hall symbol: -P 2yn	Cell parameters from 3308 reflections
*a* = 10.6685 (4) Å	θ = 2.3–29.4°
*b* = 16.8190 (5) Å	µ = 1.72 mm^−^^1^
*c* = 14.1260 (5) Å	*T* = 100 K
β = 106.303 (3)°	Prism, colorless
*V* = 2432.76 (14) Å^3^	0.20 × 0.10 × 0.05 mm
*Z* = 4	

### Data collection

**Table:**

Agilent SuperNova Dual diffractometer with an Atlas detector	5402 independent reflections
Radiation source: SuperNova (Mo) X-ray Source	3939 reflections with *I* > 2σ(*I*)
Mirror	*R*_int_ = 0.045
Detector resolution: 10.4041 pixels mm^-1^	θ_max_ = 27.5°, θ_min_ = 2.3°
ω scans	*h* = −13→13
Absorption correction: multi-scan (*CrysAlis PRO*; Agilent, 2010)	*k* = −21→19
*T*_min_ = 0.725, *T*_max_ = 0.919	*l* = −13→17
11939 measured reflections	

### Refinement

**Table:**

Refinement on *F*^2^	Primary atom site location: structure-invariant direct methods
Least-squares matrix: full	Secondary atom site location: difference Fourier map
*R*[*F*^2^ > 2σ(*F*^2^)] = 0.053	Hydrogen site location: inferred from neighbouring sites
*wR*(*F*^2^) = 0.135	H-atom parameters constrained
*S* = 1.03	*w* = 1/[σ^2^(*F*_o_^2^) + (0.0549*P*)^2^ + 1.4767*P*] where *P* = (*F*_o_^2^ + 2*F*_c_^2^)/3
5402 reflections	(Δ/σ)_max_ = 0.001
302 parameters	Δρ_max_ = 0.76 e Å^−^^3^
24 restraints	Δρ_min_ = −0.66 e Å^−^^3^

### Fractional atomic coordinates and isotropic or equivalent isotropic displacement parameters (Å^2^)

**Table:**

	*x*	*y*	*z*	*U*_iso_*/*U*_eq_	Occ. (<1)
Br1	1.00011 (3)	0.07288 (2)	0.84090 (3)	0.02822 (14)	
O1	0.6038 (2)	0.22434 (18)	1.01434 (16)	0.0322 (6)	
H1	0.5337	0.2333	0.9706	0.039*	
O2	0.3796 (2)	0.25478 (16)	0.88504 (17)	0.0306 (6)	
O3	0.7557 (3)	0.41222 (15)	0.86062 (17)	0.0284 (6)	
O4	0.6148 (10)	0.0597 (4)	0.6070 (6)	0.0224 (9)	0.636 (6)
H4	0.6748	0.0648	0.6598	0.027*	0.636 (6)
O4'	0.6669 (7)	0.0829 (4)	0.4503 (4)	0.0224 (9)	0.364
H4'	0.7173	0.0435	0.4608	0.027*	0.364 (6)
N1	0.5582 (3)	0.22319 (18)	0.61661 (19)	0.0209 (6)	
C1	0.6924 (4)	0.1925 (2)	0.9722 (2)	0.0253 (8)	
C2	0.7829 (4)	0.1395 (2)	1.0291 (2)	0.0291 (9)	
H2	0.7803	0.1273	1.0942	0.035*	
C3	0.8759 (4)	0.1044 (2)	0.9923 (2)	0.0274 (8)	
H3	0.9380	0.0686	1.0315	0.033*	
C4	0.8772 (3)	0.1223 (2)	0.8975 (2)	0.0231 (8)	
C5	0.7896 (3)	0.1759 (2)	0.8406 (2)	0.0206 (7)	
H5	0.7928	0.1873	0.7754	0.025*	
C6	0.6975 (3)	0.2134 (2)	0.8771 (2)	0.0195 (7)	
C7	0.6024 (3)	0.2741 (2)	0.8146 (2)	0.0218 (7)	
H7	0.5812	0.3146	0.8595	0.026*	
C8	0.4774 (3)	0.2344 (2)	0.7571 (2)	0.0214 (7)	
C9	0.3703 (3)	0.2291 (2)	0.8002 (2)	0.0245 (8)	
C10	0.2455 (4)	0.1933 (2)	0.7402 (3)	0.0316 (9)	
H10A	0.1952	0.2335	0.6935	0.038*	
H10B	0.1923	0.1768	0.7841	0.038*	
C11	0.2724 (4)	0.1208 (2)	0.6826 (3)	0.0320 (9)	
C12	0.3568 (3)	0.1482 (2)	0.6168 (2)	0.0260 (8)	
H12A	0.3945	0.1007	0.5937	0.031*	
H12B	0.2999	0.1750	0.5580	0.031*	
C13	0.4664 (3)	0.2038 (2)	0.6662 (2)	0.0213 (8)	
C14	0.3435 (4)	0.0568 (2)	0.7541 (3)	0.0392 (10)	
H14A	0.3604	0.0108	0.7169	0.059*	
H14B	0.2893	0.0404	0.7964	0.059*	
H14C	0.4266	0.0780	0.7951	0.059*	
C15	0.1428 (4)	0.0876 (3)	0.6174 (3)	0.0461 (12)	
H15A	0.1603	0.0414	0.5808	0.069*	
H15B	0.0980	0.1286	0.5709	0.069*	
H15C	0.0876	0.0715	0.6590	0.069*	
C16	0.5558 (3)	0.1821 (2)	0.5232 (2)	0.0253 (8)	
H16A	0.5847	0.2201	0.4799	0.030*	0.636 (6)
H16B	0.4645	0.1671	0.4893	0.030*	0.636 (6)
H16C	0.5683	0.2211	0.4740	0.030*	0.364 (6)
H16D	0.4706	0.1554	0.4958	0.030*	0.364 (6)
C17	0.6394 (8)	0.1092 (5)	0.5351 (6)	0.035 (2)	0.636 (6)
H17	0.6079	0.0794	0.4715	0.042*	0.636 (6)
C17'	0.6674 (17)	0.1202 (11)	0.5460 (9)	0.035 (2)	0.364
H17'	0.7542	0.1415	0.5846	0.042*	0.364 (6)
C18	0.7835 (8)	0.1139 (5)	0.5556 (6)	0.064 (3)	0.636 (6)
H18A	0.8061	0.1579	0.5180	0.096*	0.636 (6)
H18B	0.8165	0.0639	0.5361	0.096*	0.636 (6)
H18C	0.8230	0.1229	0.6262	0.096*	0.636 (6)
C18'	0.613 (4)	0.0541 (18)	0.598 (2)	0.064 (3)	0.364
H18D	0.6726	0.0085	0.6100	0.096*	0.364 (6)
H18E	0.5270	0.0377	0.5569	0.096*	0.364 (6)
H18F	0.6050	0.0740	0.6615	0.096*	0.364 (6)
C19	0.6444 (3)	0.2861 (2)	0.6502 (2)	0.0207 (7)	
C20	0.7162 (3)	0.3212 (2)	0.5820 (2)	0.0221 (7)	
H20A	0.6538	0.3531	0.5310	0.027*	
H20B	0.7475	0.2772	0.5480	0.027*	
C21	0.8325 (3)	0.3738 (2)	0.6323 (2)	0.0208 (7)	
C22	0.7910 (4)	0.4311 (2)	0.7019 (2)	0.0241 (8)	
H22A	0.8672	0.4633	0.7376	0.029*	
H22B	0.7236	0.4677	0.6628	0.029*	
C23	0.7371 (3)	0.3877 (2)	0.7761 (2)	0.0222 (8)	
C24	0.6607 (3)	0.3159 (2)	0.7426 (2)	0.0208 (7)	
C25	0.8732 (4)	0.4213 (2)	0.5529 (3)	0.0273 (8)	
H25A	0.9004	0.3846	0.5085	0.041*	
H25B	0.9460	0.4566	0.5845	0.041*	
H25C	0.7991	0.4531	0.5150	0.041*	
C26	0.9486 (3)	0.3234 (2)	0.6901 (2)	0.0258 (8)	
H26A	0.9746	0.2869	0.6449	0.039*	
H26B	0.9233	0.2928	0.7409	0.039*	
H26C	1.0220	0.3584	0.7214	0.039*	

### Atomic displacement parameters (Å^2^)

**Table:**

	*U*^11^	*U*^22^	*U*^33^	*U*^12^	*U*^13^	*U*^23^
Br1	0.0228 (2)	0.0251 (2)	0.0361 (2)	0.00032 (17)	0.00727 (15)	0.00592 (15)
O1	0.0261 (15)	0.0483 (19)	0.0246 (13)	−0.0083 (14)	0.0109 (10)	−0.0029 (12)
O2	0.0265 (15)	0.0350 (17)	0.0343 (14)	−0.0001 (13)	0.0150 (11)	0.0000 (12)
O3	0.0345 (15)	0.0256 (15)	0.0268 (13)	−0.0060 (12)	0.0116 (11)	−0.0040 (10)
O4	0.036 (2)	0.017 (2)	0.021 (2)	0.0144 (17)	0.0188 (16)	0.0029 (14)
O4'	0.036 (2)	0.017 (2)	0.021 (2)	0.0144 (17)	0.0188 (16)	0.0029 (14)
N1	0.0162 (15)	0.0231 (17)	0.0220 (14)	−0.0008 (13)	0.0029 (11)	0.0031 (12)
C1	0.023 (2)	0.032 (2)	0.0206 (17)	−0.0126 (17)	0.0060 (14)	−0.0035 (15)
C2	0.036 (2)	0.032 (2)	0.0185 (17)	−0.0117 (19)	0.0072 (15)	0.0053 (15)
C3	0.027 (2)	0.025 (2)	0.0243 (18)	−0.0072 (17)	−0.0018 (14)	0.0073 (15)
C4	0.0187 (19)	0.022 (2)	0.0266 (18)	−0.0048 (16)	0.0040 (14)	0.0021 (14)
C5	0.0211 (18)	0.0206 (19)	0.0192 (16)	−0.0067 (15)	0.0039 (13)	−0.0003 (13)
C6	0.0202 (19)	0.0179 (19)	0.0197 (16)	−0.0072 (15)	0.0047 (13)	−0.0003 (13)
C7	0.0217 (19)	0.0215 (19)	0.0230 (17)	−0.0027 (16)	0.0076 (13)	0.0000 (14)
C8	0.0202 (18)	0.0174 (19)	0.0258 (18)	0.0021 (15)	0.0052 (13)	0.0057 (14)
C9	0.0205 (19)	0.022 (2)	0.0307 (19)	0.0015 (16)	0.0070 (14)	0.0067 (15)
C10	0.020 (2)	0.042 (3)	0.034 (2)	−0.0015 (18)	0.0095 (15)	0.0067 (17)
C11	0.024 (2)	0.034 (2)	0.035 (2)	−0.0081 (18)	0.0030 (15)	0.0056 (17)
C12	0.0193 (19)	0.027 (2)	0.0292 (19)	−0.0010 (17)	0.0025 (14)	0.0030 (15)
C13	0.0149 (17)	0.024 (2)	0.0242 (18)	0.0024 (15)	0.0033 (13)	0.0066 (14)
C14	0.044 (3)	0.030 (2)	0.041 (2)	−0.011 (2)	0.0076 (18)	0.0081 (17)
C15	0.033 (2)	0.059 (3)	0.042 (2)	−0.023 (2)	0.0049 (18)	0.006 (2)
C16	0.0224 (19)	0.029 (2)	0.0244 (18)	0.0014 (17)	0.0072 (14)	0.0023 (15)
C17	0.031 (5)	0.042 (4)	0.038 (3)	0.010 (4)	0.020 (3)	0.016 (2)
C17'	0.031 (5)	0.042 (4)	0.038 (3)	0.010 (4)	0.020 (3)	0.016 (2)
C18	0.066 (5)	0.069 (5)	0.058 (4)	0.001 (4)	0.021 (4)	0.013 (4)
C18'	0.066 (5)	0.069 (5)	0.058 (4)	0.001 (4)	0.021 (4)	0.013 (4)
C19	0.0142 (17)	0.0214 (19)	0.0256 (17)	0.0034 (15)	0.0041 (13)	0.0065 (14)
C20	0.0202 (18)	0.023 (2)	0.0253 (17)	0.0049 (16)	0.0091 (13)	0.0072 (14)
C21	0.0193 (18)	0.0197 (19)	0.0252 (17)	0.0019 (15)	0.0092 (13)	0.0040 (14)
C22	0.028 (2)	0.0162 (19)	0.0308 (19)	0.0032 (16)	0.0136 (15)	0.0049 (14)
C23	0.0170 (18)	0.020 (2)	0.0299 (19)	0.0031 (15)	0.0075 (14)	0.0055 (14)
C24	0.0155 (17)	0.0199 (19)	0.0270 (18)	−0.0001 (15)	0.0061 (13)	0.0053 (14)
C25	0.030 (2)	0.024 (2)	0.0312 (19)	0.0009 (17)	0.0142 (15)	0.0045 (15)
C26	0.0206 (19)	0.027 (2)	0.0304 (19)	0.0010 (17)	0.0088 (14)	0.0023 (15)

### Geometric parameters (Å, °)

**Table:**

Br1—C4	1.908 (4)	C14—H14C	0.9800
O1—C1	1.360 (4)	C15—H15A	0.9800
O1—H1	0.8400	C15—H15B	0.9800
O2—C9	1.251 (4)	C15—H15C	0.9800
O3—C23	1.226 (4)	C16—C17	1.499 (7)
O4—C17	1.394 (7)	C16—C17'	1.547 (9)
O4—H4	0.8400	C16—H16A	0.9900
O4'—C17'	1.488 (10)	C16—H16B	0.9900
O4'—H4'	0.8400	C16—H16C	0.9900
N1—C13	1.393 (4)	C16—H16D	0.9900
N1—C19	1.394 (4)	C17—C18	1.484 (8)
N1—C16	1.483 (4)	C17—H17	1.0000
C1—C2	1.391 (5)	C17'—C18'	1.535 (10)
C1—C6	1.405 (5)	C17'—H17'	1.0000
C2—C3	1.376 (5)	C18—H18A	0.9800
C2—H2	0.9500	C18—H18B	0.9800
C3—C4	1.376 (5)	C18—H18C	0.9800
C3—H3	0.9500	C18'—H18D	0.9800
C4—C5	1.382 (5)	C18'—H18E	0.9800
C5—C6	1.383 (5)	C18'—H18F	0.9800
C5—H5	0.9500	C19—C24	1.364 (5)
C6—C7	1.532 (5)	C19—C20	1.509 (4)
C7—C24	1.507 (4)	C20—C21	1.526 (5)
C7—C8	1.509 (5)	C20—H20A	0.9900
C7—H7	1.0000	C20—H20B	0.9900
C8—C13	1.357 (5)	C21—C22	1.529 (5)
C8—C9	1.440 (5)	C21—C26	1.533 (5)
C9—C10	1.490 (5)	C21—C25	1.536 (5)
C10—C11	1.538 (6)	C22—C23	1.516 (5)
C10—H10A	0.9900	C22—H22A	0.9900
C10—H10B	0.9900	C22—H22B	0.9900
C11—C14	1.525 (5)	C23—C24	1.459 (5)
C11—C12	1.535 (5)	C25—H25A	0.9800
C11—C15	1.535 (5)	C25—H25B	0.9800
C12—C13	1.507 (5)	C25—H25C	0.9800
C12—H12A	0.9900	C26—H26A	0.9800
C12—H12B	0.9900	C26—H26B	0.9800
C14—H14A	0.9800	C26—H26C	0.9800
C14—H14B	0.9800		
			
C1—O1—H1	109.5	C17'—C16—H16A	102.0
C17'—O4'—H4'	109.5	N1—C16—H16B	108.6
C13—N1—C19	119.7 (3)	C17—C16—H16B	108.6
C13—N1—C16	119.9 (3)	H16A—C16—H16B	107.5
C19—N1—C16	120.1 (3)	N1—C16—H16C	110.1
O1—C1—C2	117.0 (3)	C17—C16—H16C	115.3
O1—C1—C6	122.5 (3)	C17'—C16—H16C	110.1
C2—C1—C6	120.4 (3)	N1—C16—H16D	110.1
C3—C2—C1	120.9 (3)	C17'—C16—H16D	110.1
C3—C2—H2	119.5	H16C—C16—H16D	108.4
C1—C2—H2	119.5	O4—C17—C18	106.6 (7)
C2—C3—C4	118.6 (3)	O4—C17—C16	110.0 (6)
C2—C3—H3	120.7	C18—C17—C16	122.0 (8)
C4—C3—H3	120.7	O4—C17—H17	105.7
C3—C4—C5	121.3 (3)	C18—C17—H17	105.7
C3—C4—Br1	120.5 (3)	C16—C17—H17	105.7
C5—C4—Br1	118.2 (2)	O4'—C17'—C18'	103.1 (17)
C6—C5—C4	121.0 (3)	O4'—C17'—C16	106.7 (8)
C6—C5—H5	119.5	C18'—C17'—C16	102 (2)
C4—C5—H5	119.5	O4'—C17'—H17'	114.4
C5—C6—C1	117.7 (3)	C18'—C17'—H17'	114.4
C5—C6—C7	120.9 (3)	C16—C17'—H17'	114.4
C1—C6—C7	121.4 (3)	C17—C18—H18A	109.5
C24—C7—C8	108.3 (3)	C17—C18—H18B	109.5
C24—C7—C6	111.6 (3)	H18A—C18—H18B	109.5
C8—C7—C6	110.9 (3)	C17—C18—H18C	109.5
C24—C7—H7	108.7	H18A—C18—H18C	109.5
C8—C7—H7	108.7	H18B—C18—H18C	109.5
C6—C7—H7	108.7	C17'—C18'—H18D	109.5
C13—C8—C9	120.6 (3)	C17'—C18'—H18E	109.5
C13—C8—C7	120.3 (3)	H18D—C18'—H18E	109.5
C9—C8—C7	119.0 (3)	C17'—C18'—H18F	109.5
O2—C9—C8	121.7 (3)	H18D—C18'—H18F	109.5
O2—C9—C10	120.1 (3)	H18E—C18'—H18F	109.5
C8—C9—C10	118.2 (3)	C24—C19—N1	120.0 (3)
C9—C10—C11	110.6 (3)	C24—C19—C20	121.1 (3)
C9—C10—H10A	109.5	N1—C19—C20	119.0 (3)
C11—C10—H10A	109.5	C19—C20—C21	115.0 (3)
C9—C10—H10B	109.5	C19—C20—H20A	108.5
C11—C10—H10B	109.5	C21—C20—H20A	108.5
H10A—C10—H10B	108.1	C19—C20—H20B	108.5
C14—C11—C12	110.2 (3)	C21—C20—H20B	108.5
C14—C11—C15	109.7 (3)	H20A—C20—H20B	107.5
C12—C11—C15	109.2 (3)	C20—C21—C22	108.7 (3)
C14—C11—C10	110.0 (3)	C20—C21—C26	111.0 (3)
C12—C11—C10	108.2 (3)	C22—C21—C26	110.0 (3)
C15—C11—C10	109.6 (4)	C20—C21—C25	108.8 (3)
C13—C12—C11	114.6 (3)	C22—C21—C25	109.5 (3)
C13—C12—H12A	108.6	C26—C21—C25	109.0 (3)
C11—C12—H12A	108.6	C23—C22—C21	112.1 (3)
C13—C12—H12B	108.6	C23—C22—H22A	109.2
C11—C12—H12B	108.6	C21—C22—H22A	109.2
H12A—C12—H12B	107.6	C23—C22—H22B	109.2
C8—C13—N1	120.0 (3)	C21—C22—H22B	109.2
C8—C13—C12	121.5 (3)	H22A—C22—H22B	107.9
N1—C13—C12	118.5 (3)	O3—C23—C24	121.2 (3)
C11—C14—H14A	109.5	O3—C23—C22	121.5 (3)
C11—C14—H14B	109.5	C24—C23—C22	117.4 (3)
H14A—C14—H14B	109.5	C19—C24—C23	121.6 (3)
C11—C14—H14C	109.5	C19—C24—C7	120.6 (3)
H14A—C14—H14C	109.5	C23—C24—C7	117.8 (3)
H14B—C14—H14C	109.5	C21—C25—H25A	109.5
C11—C15—H15A	109.5	C21—C25—H25B	109.5
C11—C15—H15B	109.5	H25A—C25—H25B	109.5
H15A—C15—H15B	109.5	C21—C25—H25C	109.5
C11—C15—H15C	109.5	H25A—C25—H25C	109.5
H15A—C15—H15C	109.5	H25B—C25—H25C	109.5
H15B—C15—H15C	109.5	C21—C26—H26A	109.5
N1—C16—C17	114.8 (4)	C21—C26—H26B	109.5
N1—C16—C17'	107.9 (5)	H26A—C26—H26B	109.5
C17—C16—C17'	13.0 (11)	C21—C26—H26C	109.5
N1—C16—H16A	108.6	H26A—C26—H26C	109.5
C17—C16—H16A	108.6	H26B—C26—H26C	109.5
			
O1—C1—C2—C3	180.0 (3)	C11—C12—C13—C8	−8.0 (5)
C6—C1—C2—C3	−2.3 (6)	C11—C12—C13—N1	171.6 (3)
C1—C2—C3—C4	−0.5 (6)	C13—N1—C16—C17	−91.6 (6)
C2—C3—C4—C5	1.8 (5)	C19—N1—C16—C17	94.3 (6)
C2—C3—C4—Br1	−177.9 (3)	C13—N1—C16—C17'	−103.5 (10)
C3—C4—C5—C6	−0.1 (5)	C19—N1—C16—C17'	82.4 (10)
Br1—C4—C5—C6	179.5 (3)	N1—C16—C17—O4	49.4 (8)
C4—C5—C6—C1	−2.6 (5)	C17'—C16—C17—O4	110 (4)
C4—C5—C6—C7	178.7 (3)	N1—C16—C17—C18	−76.5 (8)
O1—C1—C6—C5	−178.6 (3)	C17'—C16—C17—C18	−16 (3)
C2—C1—C6—C5	3.8 (5)	N1—C16—C17'—O4'	−177.2 (10)
O1—C1—C6—C7	0.1 (5)	C17—C16—C17'—O4'	59 (3)
C2—C1—C6—C7	−177.6 (3)	N1—C16—C17'—C18'	74.8 (16)
C5—C6—C7—C24	−28.8 (4)	C17—C16—C17'—C18'	−49 (4)
C1—C6—C7—C24	152.6 (3)	C13—N1—C19—C24	15.8 (5)
C5—C6—C7—C8	92.0 (4)	C16—N1—C19—C24	−170.1 (3)
C1—C6—C7—C8	−86.6 (4)	C13—N1—C19—C20	−163.1 (3)
C24—C7—C8—C13	34.3 (4)	C16—N1—C19—C20	11.1 (4)
C6—C7—C8—C13	−88.5 (4)	C24—C19—C20—C21	16.5 (5)
C24—C7—C8—C9	−146.0 (3)	N1—C19—C20—C21	−164.7 (3)
C6—C7—C8—C9	91.2 (4)	C19—C20—C21—C22	−46.6 (4)
C13—C8—C9—O2	177.8 (3)	C19—C20—C21—C26	74.4 (4)
C7—C8—C9—O2	−1.9 (5)	C19—C20—C21—C25	−165.8 (3)
C13—C8—C9—C10	−3.3 (5)	C20—C21—C22—C23	55.7 (4)
C7—C8—C9—C10	177.0 (3)	C26—C21—C22—C23	−65.9 (4)
O2—C9—C10—C11	−140.9 (3)	C25—C21—C22—C23	174.4 (3)
C8—C9—C10—C11	40.3 (4)	C21—C22—C23—O3	144.8 (3)
C9—C10—C11—C14	62.2 (4)	C21—C22—C23—C24	−35.9 (4)
C9—C10—C11—C12	−58.2 (4)	N1—C19—C24—C23	−172.3 (3)
C9—C10—C11—C15	−177.2 (3)	C20—C19—C24—C23	6.5 (5)
C14—C11—C12—C13	−77.2 (4)	N1—C19—C24—C7	9.1 (5)
C15—C11—C12—C13	162.3 (3)	C20—C19—C24—C7	−172.1 (3)
C10—C11—C12—C13	43.1 (4)	O3—C23—C24—C19	−177.0 (3)
C9—C8—C13—N1	166.7 (3)	C22—C23—C24—C19	3.7 (5)
C7—C8—C13—N1	−13.6 (5)	O3—C23—C24—C7	1.6 (5)
C9—C8—C13—C12	−13.7 (5)	C22—C23—C24—C7	−177.7 (3)
C7—C8—C13—C12	165.9 (3)	C8—C7—C24—C19	−31.9 (4)
C19—N1—C13—C8	−13.5 (5)	C6—C7—C24—C19	90.4 (4)
C16—N1—C13—C8	172.4 (3)	C8—C7—C24—C23	149.4 (3)
C19—N1—C13—C12	166.9 (3)	C6—C7—C24—C23	−88.2 (4)
C16—N1—C13—C12	−7.2 (5)		

### Hydrogen-bond geometry (Å, °)

**Table:**

*D*—H···*A*	*D*—H	H···*A*	*D*···*A*	*D*—H···*A*
O1—H1···O2	0.84	1.78	2.621 (4)	176
C10—H10A···O1^i^	0.99	2.54	3.417 (5)	147

Symmetry codes: (i) *x*−1/2, −*y*+1/2, *z*−1/2.
